# *Mycobacterium tuberculosis* Infection Induces HDAC1-Mediated Suppression of *IL-12B* Gene Expression in Macrophages

**DOI:** 10.3389/fcimb.2015.00090

**Published:** 2015-12-02

**Authors:** Aneesh Chandran, Cecil Antony, Leny Jose, Sathish Mundayoor, Krishnamurthy Natarajan, R. Ajay Kumar

**Affiliations:** ^1^Mycobacterium Research Group, Tropical Disease Biology, Rajiv Gandhi Centre for BiotechnologyThiruvananthapuram, India; ^2^Infectious Diseases Laboratory, Dr. B. R. Ambedkar Centre for Biomedical Research, University of DelhiDelhi, India

**Keywords:** host-pathogen, epigenetic modifications, CREB, THP-1 macrophages, c-jun, interleukin-12

## Abstract

Downregulation of host gene expression is one of the many strategies employed by intracellular pathogens such as *Mycobacterium tuberculosis* (MTB) to survive inside the macrophages and cause disease. The underlying molecular mechanism behind the downregulation of host defense gene expression is largely unknown. In this study we explored the role of histone deacetylation in macrophages in response to infection by virulent MTB H37Rv in manipulating host gene expression. We show a significant increase in the levels of HDAC1 with a concomitant and marked reduction in the levels of histone H3-acetylation in macrophages containing live, but not killed, virulent MTB. Additionally, we show that HDAC1 is recruited to the promoter of *IL-12B* in macrophages infected with live, virulent MTB, and the subsequent hypoacetylation of histone H3 suppresses the expression of this gene which plays a key role in initiating Th1 responses. By inhibiting immunologically relevant kinases, and by knockdown of crucial transcriptional regulators, we demonstrate that protein kinase-A (PKA), CREB, and c-Jun play an important role in regulating HDAC1 level in live MTB-infected macrophages. By chromatin immunoprecipitation (ChIP) analysis, we prove that *HDAC1* expression is positively regulated by the recruitment of c-Jun to its promoter. Knockdown of *HDAC1* in macrophages significantly reduced the survival of intracellular MTB. These observations indicate a novel HDAC1-mediated epigenetic modification induced by live, virulent MTB to subvert the immune system to survive and replicate in the host.

## Introduction

*Mycobacterium tuberculosis* (MTB) is the causative agent of tuberculosis (TB) in humans and is responsible for approximately 1.4 million deaths a year across the world (World Health Organization, 2012). The success of MTB relies in part on its ability to invade and thrive inside the macrophages of the host. This is achieved by preventing acidification and maturation of phagosome thereby evading killing by professional phagocytes (Philips, 2008). A fundamental step in eukaryotic gene expression and repression is the exposure or concealment of regulatory DNA sequences to transcriptional regulators. These two processes are dependent on highly specific, reversible covalent post-translational modifications of histones by chromatin modifying enzymes, or on physical rearrangement of nucleosomes by chromatin remodeling factors. The status of acetylation of histones plays a key role in gene expression and is maintained by two classes of enzymes, histone acetyltransferases (HATs) and histone deacetylases (HDACs) (Garcia-Garcia et al., 2009a). Chromatin modifications such as acetylation, methylation, and phosphorylation of histones are employed by intracellular pathogens to survive and cause disease in their host. For example, viruses such as adenovirus, Epstein-Barr virus, and SV40 are known to exploit various chromatin modifications to regulate transcription in the host cell in order to manipulate cellular functions to promote infection (Radkov et al., 1999; Punga and Akusjärvi, 2000; Valls et al., 2007). Observation that intracellular bacteria can manipulate host defense gene expression by epigenetic modifications to facilitate infection and survival inside the host cell is relatively recent (Arbibe et al., 2007; Hamon et al., 2007; Hamon and Cossart, 2008; Garcia-Garcia et al., 2009a,b; Marr et al., 2014), and the strategies employed by the pathogens to achieve this are not yet fully unraveled. A number of host genes, many of which are implicated in the production of molecules involved in host immune responses, are positively or negatively regulated by MTB upon infection (van Crevel et al., 2002; Hossain and Norazmi, 2013).

As MTB infection is known to cause changes in the host gene expression, we expected that at least one mechanism behind this phenomenon could be histone modification. Downregulation of immune responses is an important consequence of MTB infection. Therefore, we wished to explore how MTB infection brings about the downregulation of immune responses by histone modifications with the aid of HDACs, a class of enzymes responsible for repression of gene expression. Besides we tried to elucidate the signaling pathway in regulating histone deacetylation process in macrophages upon MTB infection, an area which has not been explored in detail.

Here we report that infection of macrophages by live virulent MTB induces upregulation of HDAC1 expression which in turn is effectively employed to deacetylate histone H3 at the promoter of *IL-12B* gene. This leads to the downregulation of expression of *IL-12B* whose product plays a crucial role in initiating Th1 immune response in the host. In addition we show that HDAC1 is upregulated through signal transduction pathway involving at least PKA, CREB, and c-Jun.

## Materials and methods

### Bacterial cultures

Virulent MTB strain H37Rv and the avirulent strain H37Ra were used in the study. All experiments with MTB were performed in a Biosafety Level III facility for the safe handling of the bacilli. Bacteria were grown in Middlebrook 7H9 broth (Difco) supplemented with 10% OADC (Becton Dickinson), 0.4% glycerol and 0.05% Tween 80. Bacteria from mid-log culture were harvested by centrifugation, washed with RPMI containing 10% FBS, and resuspended in the same medium. The suspension was dispersed by aspirating five times through a 24-gauge needle, followed by 10 times through a 30-gauge needle. This was then vortexed until no bacterial clumps were detectable, and the suspension was allowed to stand for 5 min. The upper half of the suspension was then used for the experiments. Bacteria were quantified by measuring the absorbance at a wavelength of 600 nm (0.15 O.D. corresponds to ~300 × 10^6^ bacteria). Heat-killed bacteria were prepared by incubating bacterial culture at 80°C for 30 min in a water bath.

### Infection of THP-1-derived macrophage cells with *M. tuberculosis* H37Rv

THP-1 cells were cultured in RPMI 1640 supplemented with 10% FBS, 2 mM L-glutamine, 25 mM HEPES, 1.5 g/L sodium bicarbonate, and incubated at 37°C in the presence of 5% CO_2_. Cells (3 × 10^6^/ml) were passaged in complete RPMI containing phorbol 12-myristate 13-acetate (PMA, Sigma-Aldrich) at a final concentration of 20 ng/ml, and plated for differentiation into macrophages. After 24 h, supernatants were removed and the cells were infected with live and killed MTB at a multiplicity of infection (MOI) of 20:1 and incubated for 4 h at 37°C in the presence of 5% CO_2_ for phagocytosis. Cells were then washed four times with complete RPMI containing gentamycin (10 μg/mL) to remove extracellular bacilli and fresh medium was added, followed by incubation for appropriate duration at 37°C in the presence of 5% CO_2_. For experiments in which inhibitors were used, specific inhibitors were added after phagocytosis. Final concentrations of the inhibitors were: TSA, 45 nmol/ml and SB, 2 mmol/ml; Calphostin-c, 0.1 μmol/ml; H89, 0.1 μmol/ml; U0126, 10 μmol/ml; and Wortmannin, 100 nmol/ml. The concentration of LPS used was 50 ng/ml.

Primary macrophages derived from peripheral blood mononuclear cells (PBMC, Stem Cell Technologies, Canada) were also used for infection. To isolate macrophages total PBMCs were plated in a 24-well plate and incubated for 2 h at 37°C in 5% CO_2_. Non-adherent cells were removed by washing the wells three times with basal RPMI-1640 medium. Adherent monocytes were cultured in complete RPMI medium and the cells were incubated for 4 days at 37°C in 5% CO_2_ to spontaneously differentiate them into macrophages. After 4 days, supernatants were removed and the cells were infected with live MTB as described earlier.

### Antibodies and reagents

Rabbit anti-HDAC1 was purchased from Diagenode (Belgium), rabbit anti-H3 and rabbit anti-H3Ac were obtained from Abcam (Cambridge, UK). All primary antibodies were used according to the manufacturer's instructions. Horseradish peroxidase-conjugated secondary antibodies were from Sigma-Aldrich and enhanced chemiluminescence (ECL) reagents were from Amersham Biosciences (Piscataway, USA). All other reagents were purchased from Sigma-Aldrich, unless otherwise stated.

### Transfection of macrophages with siRNA and infection

Two million cells were transfected with 60 pmol of siRNA for 36 h using the Hiperfect transfection reagent (Qiagen, Valencia, USA) in Opti-MEM medium (Invitrogen, Carlsbad, USA). Knockdown was verified by qPCR or western blot. Cells transfected with siRNA were infected with MTB H37Rv at an MOI of 20:1. After appropriate incubation period, RNA was isolated from the cells for qPCR, and the whole cell lysate was used for western blotting. Scrambled siRNA was used as control. All siRNAs were purchased from Santa Cruz Biotechnologies (Santa Cruz, USA).

### Confocal microscopy

THP-1 cells were grown on glass cover slips and induced with PMA. After 24 h the macrophages were infected with live or heat-killed MTB H37Rv, and incubated for 4 h to allow phagocytosis. Cells were then washed four times with complete RPMI medium containing gentamycin (10 μg/mL) to remove extracellular bacilli, and fresh medium was added followed by incubation for appropriate duration at 37°C in the presence of 5% CO_2_. Cells were fixed and the plasma membranes were permeabilized by incubating the cells in 1 ml of a mixture of acetone-methanol (1:1) for 20 min at −20°C, then blocked with PBS supplemented with 2% (w/v) BSA, 10% (v/v) FBS, and 1% (v/v) Triton X-100. Samples were incubated with appropriate primary antibody overnight at 4°C, washed, and incubated with FITC-conjugated secondary antibody (Sigma-Aldrich) or dyLight-conjugated secondary antibody (Abcam, UK) for 1 h at room temperature. DAPI (1 μg/ml) was added and incubated for 1 min. After washing, the cover-slips were mounted using mounting medium and the cells were visualized under a Nikon-A1 R confocal microscope at appropriate wavelengths.

### Chromatin immunoprecipitation (ChIP)

ChIP was performed according to the manufacturer's protocol (Abcam, UK). Briefly, macrophages were plated in equal densities of 3 × 10^6^ cells/ml and infected with live and heat-killed MTB H37Rv. Uninfected macrophages served as control. Macrophage cells were treated with formaldehyde (1% final concentration) in PBS for 10 min at 37°C to crosslink histones to DNA, and were washed twice with PBS (1X) containing protease inhibitor cocktail (Sigma-Aldrich). Cells were lysed and the chromatin was sheared by sonication (Bioruptor, Diagenode, Belgium) and centrifuged at 14,000 rpm for 15 min at 4°C. Chromatin fragments in the supernatant were immunoprecipitated with antibodies against multiacetylated histone H3 (Ac-H3—K9, K18, K23, K27), unacetylated histone H3, or IgG using protein A sepharose beads (Abcam, UK) overnight at 4°C on a Mini LabRoller™ Rotator (Labnet International, USA). The immunoprecipitates were washed twice with wash buffer, DNA was eluted off the beads, and the purified DNA was subjected to PCR. Primers used to amplify promoters of specific genes are listed in Table 1 in Supplementary Material. Experimentally validated promoters were selected from eukaryotic promoter database (EPD—http://epd.vital-it.ch/).

### Histone extraction and analysis

Histones were isolated from MTB-infected, heat-killed MTB-treated and uninfected cells by EpiSeeker histone extraction kit (ab113476, Abcam) as described by the manufacturer. To study changes in histone acetylation, equal amounts (1 μg) of isolated histones were separated on a 15% SDS-polyacrylamide gel and analyzed by immunoblotting using antibodies specific for Ac-H3.

### Western blotting for specific molecules

Macrophages were infected with MTB (MOI 20:1), and nuclear extracts were prepared using Nuclear Extraction Kit (Bio-Rad Laboratories, Hercules, USA). Protein concentration was determined using bicinchoninic protein assay kit (Thermo Fisher Scientific, Rockford, IL). Proteins were resolved on SDS-polyacrylamide (12%) gels, and were transferred to methanol-activated Immobilon-P polyvinylidene difluoride membranes (Millipore, Billerica, MA) and probed with specific primary antibody. Following incubation with HRP-conjugated secondary antibody, the proteins were detected by chemiluminescence and quantified densitometrically using imageJ software. Bands were normalized to intensity of corresponding bands for Histone-H3.

### Quantitative real-time PCR (qPCR)

After infection, total RNA was isolated from macrophages using illustra RNAspin Mini Kit (GE Healthcare, Piscataway, USA) according to the manufacturer's protocol and qPCR was carried out following MIQE guidelines (Bustin et al., 2009). One microgram of the RNA was converted to cDNA using the GoScript™ Reverse Transcription System (Promega, Madison, WI). The cDNA was subjected to qPCR using the iQTM SYBR® Green Supermix (Bio-Rad Laboratories, Hercules, CA) on an iCycler iQ™ Real-Time PCR Detection System (Bio-Rad). Melt-curve analysis was performed to confirm that the signal was of the expected amplification product. The values for specific genes were normalized to human *GAPDH*, which was found to be working well with our sample after analysing a set of housekeeping genes in our experimental system. The primers used for qPCR are listed in Table 2 in Supplementary Material. All qPCR primers were purchased from Sigma-Aldrich.

### ELISA

Cell supernatants of infected macrophages were collected, filtered, and the quantity of IL-12p40 was determined using an ELISA kit (BD OpteEIA™ Human IL-12(p40) kit, BD Biosciences, USA) according to the manufacturer's instructions.

### Survival assay for intracellular MTB

The human macrophage cell line, THP-1 was maintained in RPMI 1640 medium (Sigma-Aldrich, MO, USA) supplemented with 10% fetal bovine serum (Gibco, NY, USA) at 37°C in a 5% CO_2_ humidified atmosphere. 1 × 10^6^ cells were seeded per well into 6-well plates (Corning, NY, USA) and treated with PMA. Infection of the macrophages was carried out as mentioned earlier. After 4 h, the infected cells were washed twice with warm RPMI to remove non-phagocytosed bacteria followed by the addition of 1 ml of medium with 10 μg/ml gentamycin (HiMedia, Mumbai, India). After incubation for 2 h, the cells were washed twice with PBS and then maintained in complete RPMI for the rest of the experiment. Cells were processed at 24, 48, and 72 h after infection. The macrophage monolayers were washed with PBS, lysed with 500 μl 0.06% SDS in Middlebrook 7H9 medium and the released bacteria pelleted and resuspended in 100 μl of the same. The intracellular bacteria were quantified by plating them on solid agar plates to determine colony forming units (cfu). The experiment was performed in triplicates.

Determination of cfu: 20 μl of the cell suspension was plated on Middlebrook 7H10 agar plates and incubated at 37°C. After 3 weeks, the bacterial colonies on plates were counted.

### Statistical analysis

All statistical analyses were performed with GraphPad Prism® software (GraphPad Prism version 5.01 for Windows; GraphPad software, San Diego, CA, USA), and the data were expressed as mean values with their standard deviations. The results were analyzed by non-parametric analysis of variance (ANOVA), followed by Boneferroni's multiple comparison test. A *p* < 0.05 was considered significant.

## Results

### Levels of HDAC1 increase in macrophages upon MTB infection

**Figure 1:**
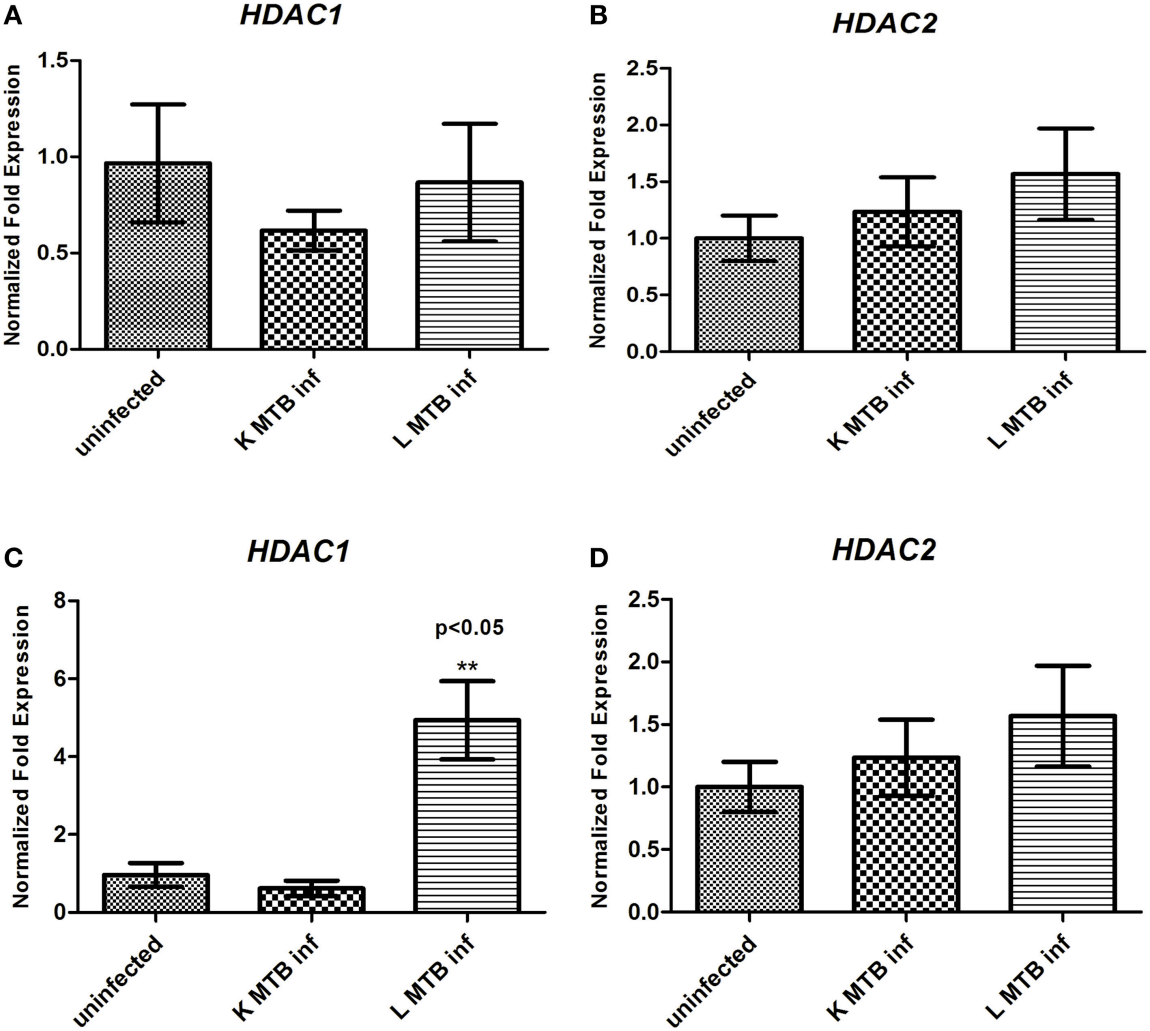
**Status of expression of ***HDAC1*** and ***HDAC2*** in macrophages upon MTB infection. (A)** *HDAC1* and **(B)** *HDAC2* expression at 12 h; **(C)** *HDAC1* and **(D)** *HDAC2* expression at 24 h. Each result represents the mean ± SD of data from three experiments. ^**^p < 0.05.

**Figure 2:**
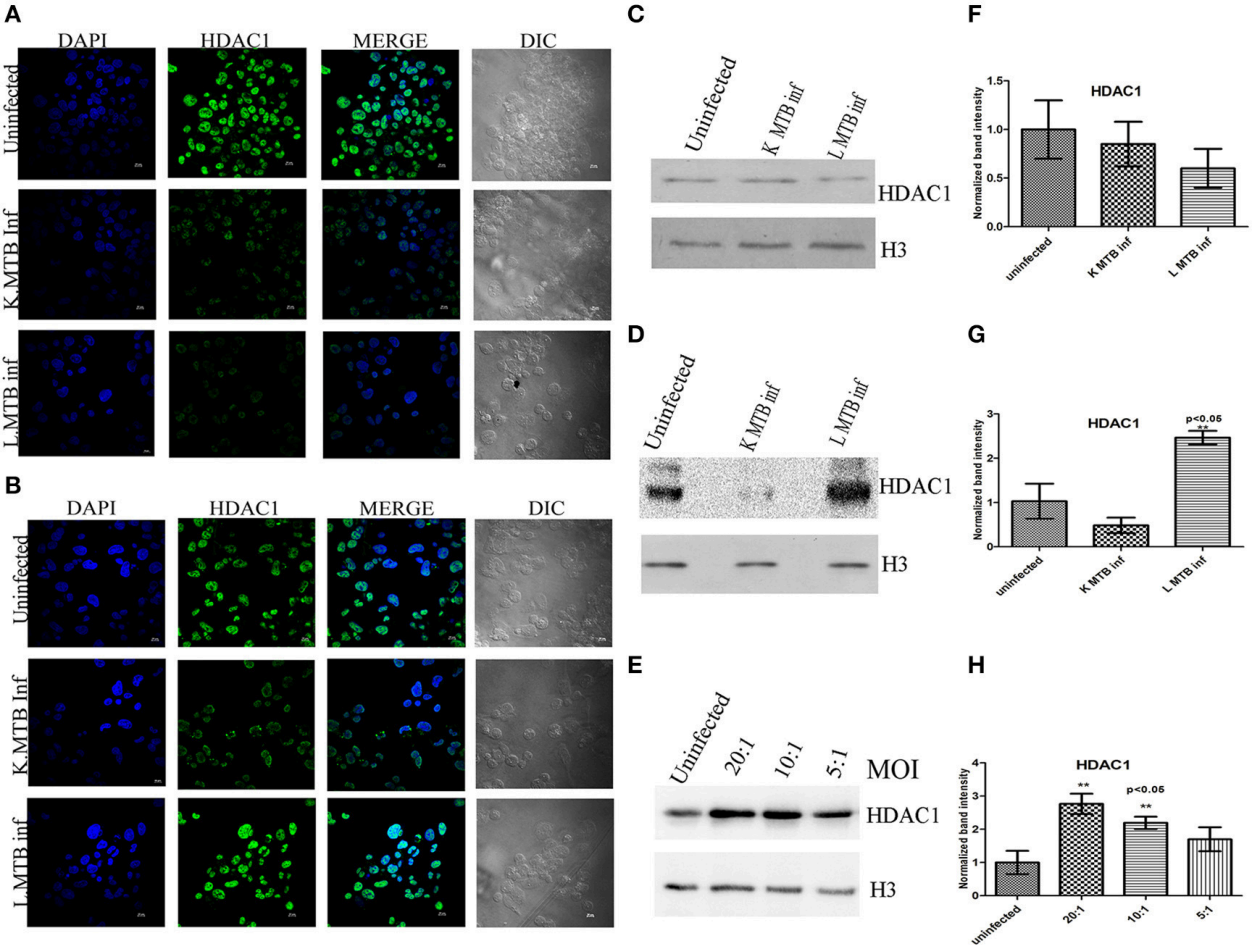
**Differential expression of HDAC1 upon MTB infection**. **(A)** Immuno-cytochemical imaging shows expression of HDAC1 in variously infected macrophages at 12 h; **(B)** 24 h post infection (PI); **(C)** at 12 h PI, levels of HDAC1 are low in MTB-infected macrophage cells; **(D)** at 24 h PI, levels of HDAC1 are high in MTB-infected macrophages; **(E)** levels of HDAC1 expression depend on the bacterial load inside the macrophages at 24 h PI; **(F–H)** densitometric analysis of HDAC1 bands normalized with that of histone H3. Quantitative data of HDAC1 protein is expressed as the mean of ratio of densitometric values of HDAC1 to histone H3 bands ± SD for three independent experiments. **p < 0.05.

Hypoacetylation of histones on the promoters is a well-known mechanism to downregulate gene expression in eukaryotes (Jenuwein and Allis, 2001; Roth et al., 2001). To delineate the transcript level of HDACs during MTB infection, we measured the levels of *HDAC1* and *HDAC2* expression in human macrophages following infection. Toward this, we infected macrophages with live and heat-killed MTB H37Rv, isolated total RNA at 12 h and 24 h post-infection (PI), and carried out a qPCR for these genes. We observed that at 12 h PI, *HDAC1* and *HDAC2* were marginally downregulated in macrophages infected with live, virulent MTB (Figures 1A,B) whereas at 24 h PI, the expression of *HDAC1* was found to be significantly upregulated by about five-fold (Figure 1C), and no marked change was observed in *HDAC2* expression (Figure 1D) when compared to uninfected macrophages and those infected with heat-killed MTB. To compare HDAC1 at the protein level, we carried out immunocytochemical imaging and observed that at 12 h, the fluorescence signal for HDAC1 from macrophages infected with live, virulent MTB was equal to that from macrophages infected with heat-killed MTB, but lower than that from uninfected macrophages (Figure 2A). Surprisingly at 24 h PI, the fluorescence signal for HDAC1 from macrophages infected with live MTB was stronger than that in macrophages infected with heat-killed MTB (Figure 2B). The mean fluorescence intensity was calculated to confirm this observation (Figure S1). To confirm that the high level of HDAC1 is due to the presence of live intracellular bacteria, we infected the cells with MTB expressing GFP (Figure S2). We carried out a western blot analysis of nuclear proteins from uninfected, live and heat-killed MTB-infected macrophages at 12 h and 24 h PI (Figures 2C,D). At 12 h PI we observed a decrease in HDAC1 protein level in macrophages infected with live as well as heat-killed MTB (Figures 2C,F). However, at 24 h PI, the HDAC1 level increased by about 2.4 fold in live, virulent MTB-infected macrophages, and decreased by about 0.4 fold in macrophages infected with heat-killed MTB (Figures 2D,G). The levels of HDAC1 in macrophages treated with LPS or infected with non-virulent MTB H37Ra remained unaltered (Figure S3). To investigate whether HDAC1 expression was dependent on the strength of infection, we infected macrophages with live MTB H37Rv at different MOI, and HDAC1 was quantified at 24 h PI. HDAC1 was found to increase as a function of the number of intracellular bacteria up to an MOI of 20:1 (Figures 2E,H).

### Kinetics of HDAC1 in macrophages upon MTB infection

**Figure 3:**
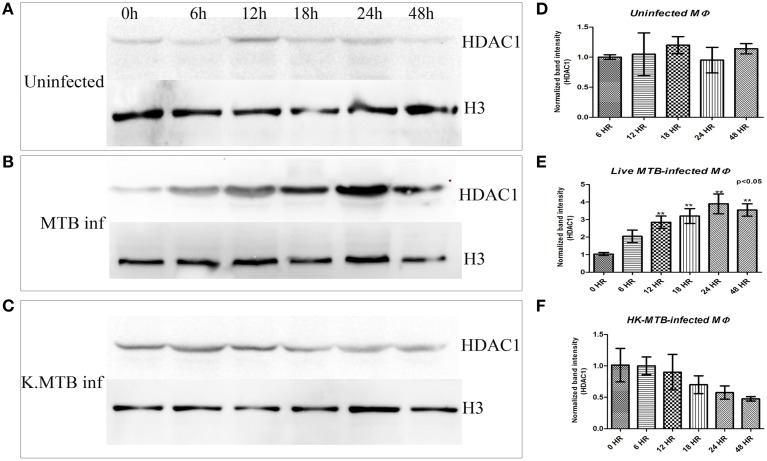
**Kinetics of HDAC1 in macrophages upon MTB infection. (A)** No significant change in the levels of HDAC1 in uninfected macrophages at 0, 6, 12, 18, 24, and 48 h PI, **(B)** HDAC1 level increases and peaks at 24 h in the case of macrophages infected with live, virulent MTB, **(C)** HDAC1 level drops gradually in macrophages containing heat-killed MTB. **(D–F)** Densitometric analysis of HDAC1 bands normalized with histone H3. Quantitative data of HDAC1 protein is expressed as the mean of ratio of densitometries of HDAC1 to histone H3 bands ± SD for three independent experiments. Levels of HDAC1 protein increased significantly (^**^P < 0.05 vs. 0 h) only in the case of macrophages infected with live, virulent MTB.

Kinetics of HDAC1 in uninfected macrophages and macrophages infected with live and heat-killed MTB was analyzed by western blot of HDAC1 at different time points. The blot clearly shows that the signal gradually increased only in macrophages infected with live virulent MTB, peaking at 24 h (Figures 3B,E) whereas in the case of macrophages treated with heat-killed bacteria, the levels dropped as time increased after infection (Figures 3C,F). There was no significant change in the levels of HDAC1 in uninfected macrophages (Figures 3A,D).

### Histone H3 acetylation decreases in macrophages upon MTB infection

**Figure 4:**
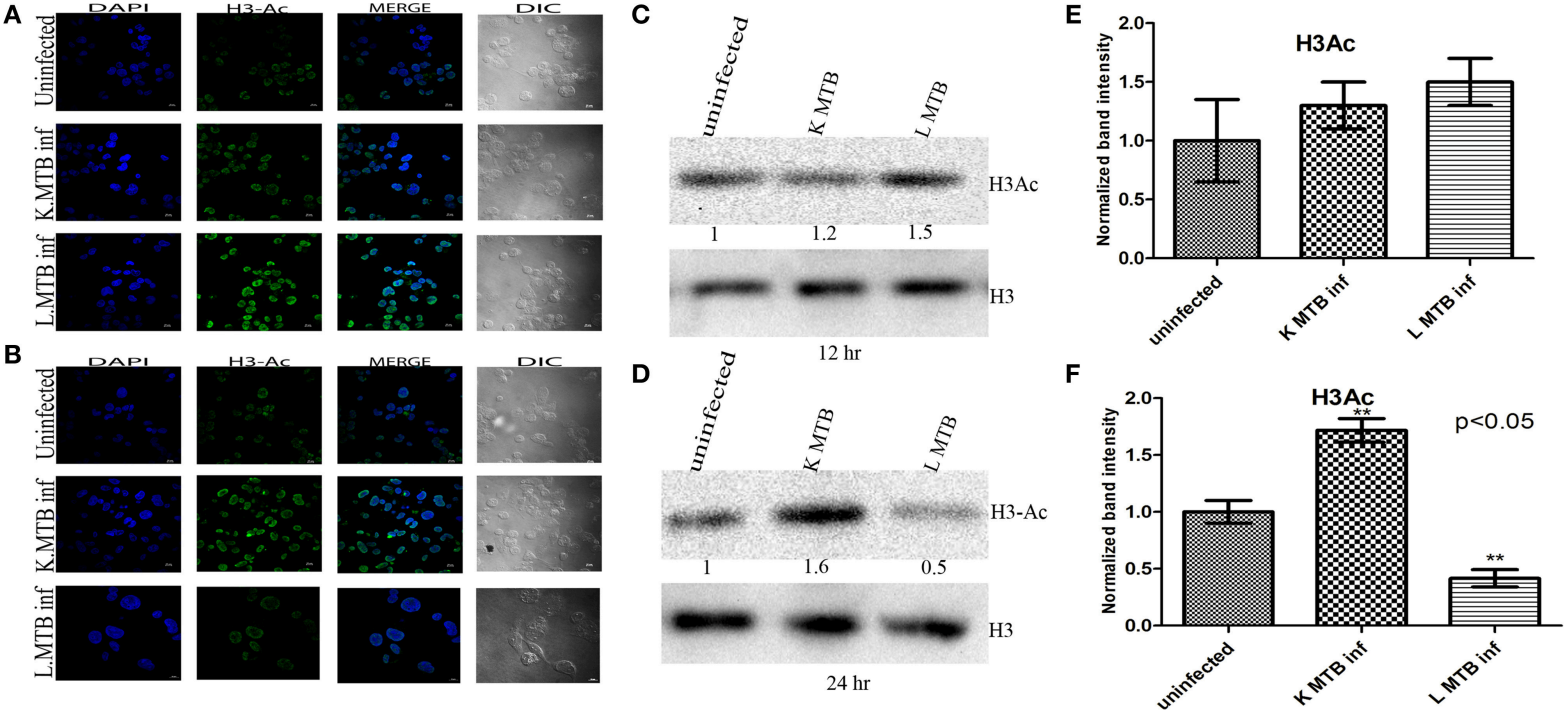
**Histone H3 acetylation status in macrophages upon MTB infection. (A)** Increase in histone H3-acetylation status in MTB-infected macrophages at 12 h PI is visualized by confocal microscopy; **(B)** decrease in histone H3-acetylation in MTB-infected macrophages at 24 h PI; **(C,D)** different levels of acetylated histone H3 at 12 h and 24 h PI by western blot. **(E,F)** Densitometric analysis of H3Ac bands normalized with histone H3. Quantitative data of HDAC1 protein is expressed as the mean of ratio of densitometries of HDAC1 to histone H3 bands ± SD for three independent experiments. ^**^p < 0.05.

To investigate whether differential expression of HDAC1 observed at 12 h and 24 h post infection inversely correlated with the acetylation status of histones; we extracted histones and then analyzed the levels of histone H3 acetylation in macrophages by blotting, and carried out an immunocytochemical imaging in parallel. Histone modifications such as hyperacetylation of histone H3 is known to be an active marker of gene expression and are often associated with ongoing transcription (Roh et al., 2005). We observed that MTB infection led to an increase in total histone H3-acetylation in macrophages at 12 h PI (Figures 4A,C,E), whereas we observed a marked decrease of the same at 24 h PI (Figures 4B,D,F) compared to the levels in uninfected cells and those infected with killed MTB. This indicated that the increase in HDAC1 levels upon infection with live MTB H37Rv corroborated well with a concomitant decrease in histone H3 acetylation.

### Recruitment of HDAC1 and subsequent histone H3 deacetylation on the promoter of *IL-12B* gene in macrophages infected with live MTB H37Rv

**Figure 5:**
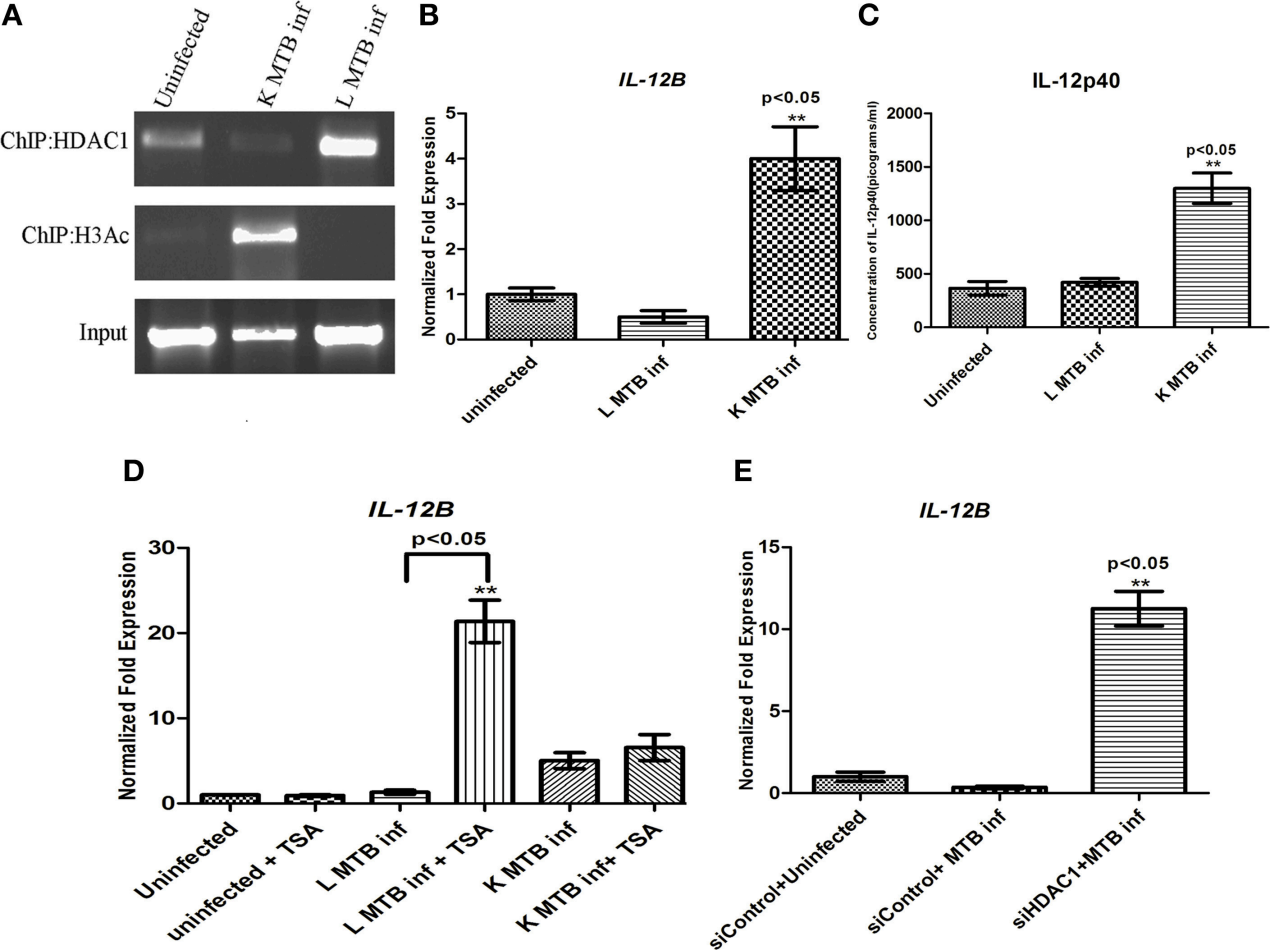
**HDAC1 downregulates the ***IL-12B*** expression in macrophages 24 h post-MTB infection. (A)** Status of HDAC1 and H3-Ac on the *IL12B* promoter by ChIP; **(B)** qPCR of *IL-12B;* **(C)** ELISA of IL-12p40. **(D)** *IL-12* expression is upregulated in MTB-infected macrophages upon HDAC inhibition; **(E)** *IL-12* expression is upregulated in MTB-infected macrophages when HDAC1 is knocked down. One of three independent experiments is shown for **(A)**; for **(B–E)** data are the mean ± SD of three independent experiments. ^**^p < 0.05.

Intracellular pathogens upon infection elicit Th1 responses at the sites of infection (D'elios et al., 2011). As IL-12p40, the product of *IL-12B* gene, is an important Th1 regulator (Romani et al., 1994; Cooper et al., 1997) we performed chromatin immunoprecipitation (ChIP) to find out if HDAC1 binds to the promoter region of this gene and plays a role in regulating its expression in macrophages during MTB infection. We observed that following infection with live MTB H37Rv at 24 h PI, there was an increased recruitment of HDAC1 to the promoter of IL-*12B* gene (Figure 5A). The levels of histone H3-acetylation on *IL-12B* promoter was found to decrease as a consequence of infection with live, virulent MTB (Figure 5A). Subsequently by qPCR we demonstrated that this gene is downregulated under the same conditions (Figure 5B). Interestingly this gene was found to be significantly upregulated in macrophages containing heat-killed MTB H37Rv. To estimate the levels of IL-12p40, an ELISA was carried out with the cell supernatants obtained from different infection conditions at 24 h PI. IL-12p40 levels in macrophages infected with live MTB did not show any significant change, whereas cells containing killed MTB showed a significant increase in its level compared to uninfected macrophages at 24 h PI (Figure 5C).

### Knockdown of *HDAC1* reverses the expression of *IL-12B* in MTB-infected macrophages

The above observations that HDAC1 can bring about the downregulation of *IL-12B* gene in infected macrophages suggested that it should be possible to reverse this downregulation upon HDAC1 inhibition. To verify this, we treated macrophages with HDAC inhibitor Trichostatin-A (TSA) (Dokmanovic et al., 2007), and infected the cells with live, virulent MTB. The cells treated with TSA and infected with live MTB showed an upregulation of *IL-12B* (Figure 5D) whereas there was no significant change in the status of expression of these genes in uninfected macrophages or those infected with killed MTB H37Rv. Sodium butyrate (SB), another HDAC inhibitor, also showed a similar effect albeit to a smaller extent (Figure S4A). Since TSA and SB are not specific inhibitors of HDAC1, we knocked down *HDAC1* with siRNA to check if we would observe the same effect. As expected, knockdown of *HDAC1* significantly increased the levels of *IL-12B* expression (Figure 5E) in macrophages infected with live MTB, thus confirming the observation that downregulation of IL-12p40 upon infection with live MTB was indeed a result of infection-induced HDAC1 expression. Knockdown of *HDAC2* did not show any significant change in the level of IL-12B in macrophages upon MTB infection (Figure S4B). Efficiency and specificity of siRNA-mediated knock down of *HDAC1* was also confirmed by qPCR and western blot (Figures S5A,B).

### Status of HDAC1 and IL-12p40 in peripheral blood mononuclear cells upon MTB infection

**Figure 6:**
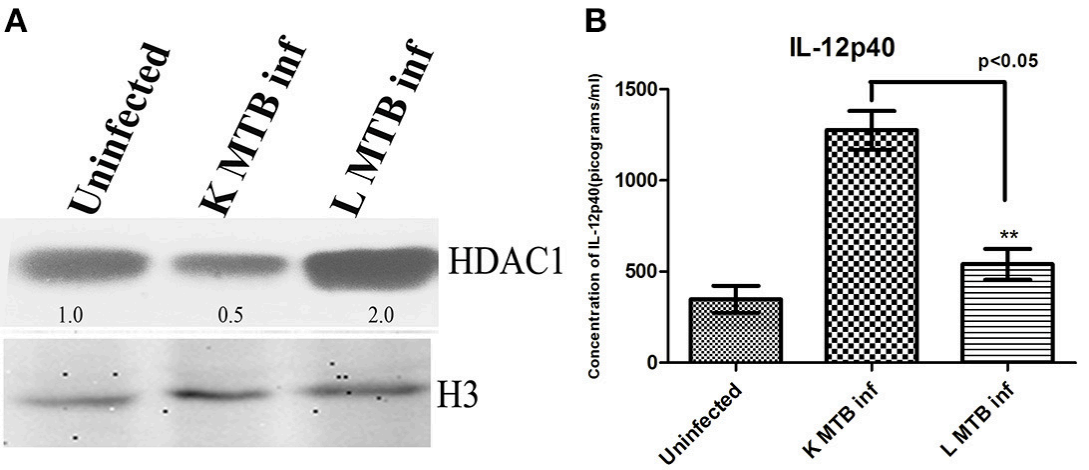
**HDAC1 and IL-12p40 status in PBMC-derived macrophages upon MTB infection. (A)** HDAC1 level increases in macrophages upon infection with live virulent MTB **(B)** Levels of IL-12p40 are significantly low in macrophages infected with live virulent MTB, compared to those cells treated with killed bacteria. One of three independent experiments is shown in case of **(A)**; for **(B)** data are the mean ± SD of three independent experiments. The numbers indicated below the blots are the normalized densitometry values of the bands calculated by imageJ software. ^**^p < 0.05.

To test the status of HDAC1 levels in primary macrophage cells upon MTB infection, we infected macrophages derived from PBMCs with MTB. We observed an increase of ~2.0 fold in the level of HDAC1 (Figure 6A). Similarly, consistent with our observation in THP-1 derived macrophages, the level of IL-12p40 was found to be significantly low in macrophages infected with live virulent MTB, compared to those cells treated with killed bacteria (Figure 6B).

### HDAC1 levels in MTB-infected macrophages are dependent on protein kinase-A activity

**Figure 7:**
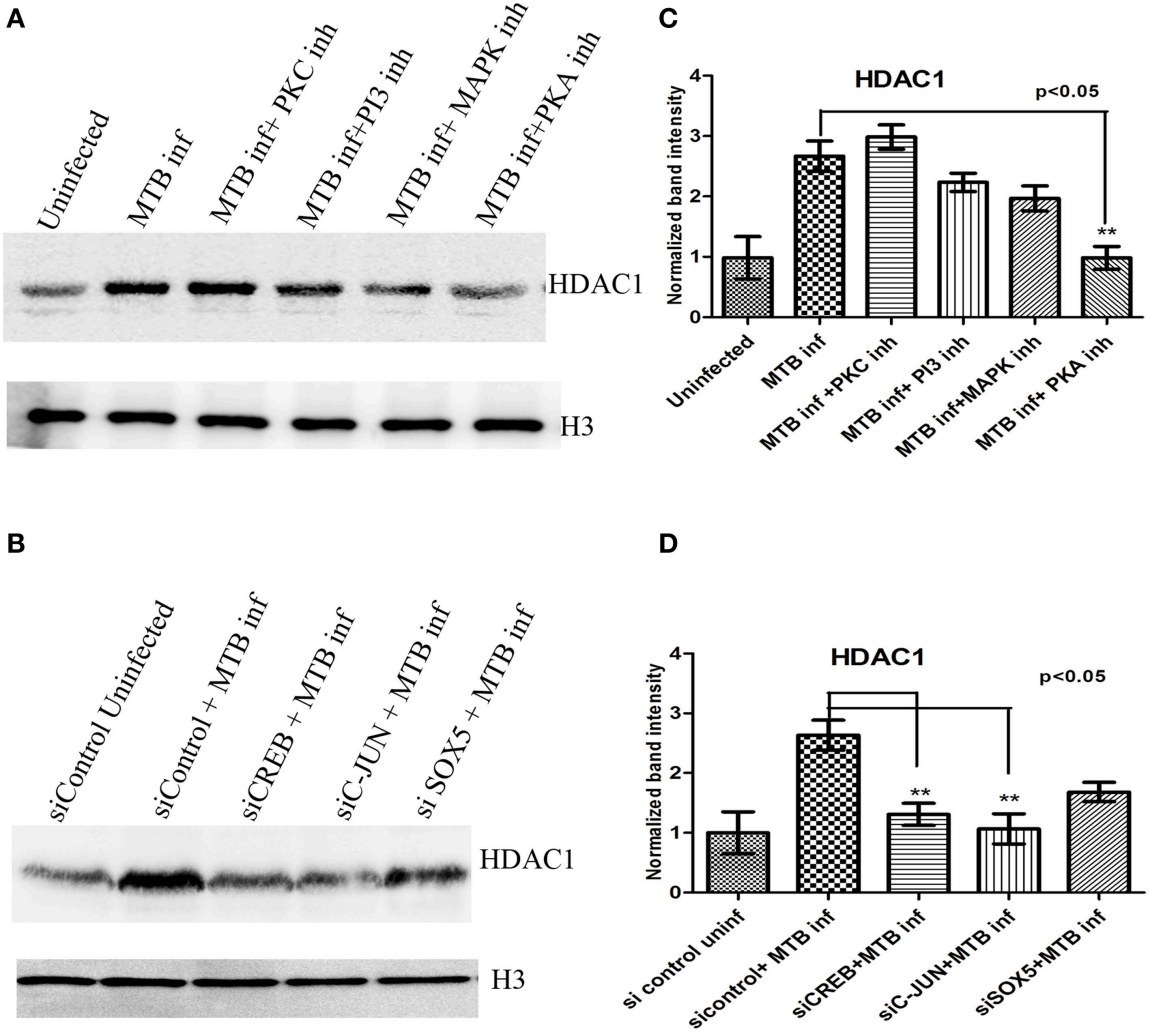
**HDAC1 levels in macrophages upon MTB infection in the presence of kinase inhibitors, and after knock-down of transcriptional regulators. (A)** HDAC1 levels are significantly low when PKA is inhibited, **(B)** HDAC1 levels are significantly low when *CREB* and *C-jun* are knocked down and a marginal difference is observed in the case of macrophages in which SOX5 is knocked down. **(C,D)** Densitometric analysis of HDAC1 bands normalized with that of histone H3. Quantitative data of HDAC1 protein is expressed as the mean of ratio of densitometric values of HDAC1 to histone H3 bands ± SD for three independent experiments. ^**^p < 0.05.

Although the role of HDACs in the regulation of gene expression is well studied (Delcuve et al., 2012; Winter et al., 2013; Kumar et al., 2014), the regulation of expression of HDAC itself is sparsely understood, especially during bacterial infections. Professional phagocytes, in response to their encounter with microorganisms, initiate immune reactions through signal transduction pathways which principally depend on the phosphorylation and dephosphorylation of the participating molecules by kinases and phosphatases. Many protein kinases play a major role in signal transduction cascades (Hanks and Hunter, 1995). For example, Mitogen-activated_Protein Kinase (MAPK-ERK) has been shown to play an important role in proinflamatory immune responses (Liu et al., 2007). Protein Kinase-C (PKC) has a role in protection against both intracellular and extracellular pathogens by activating other kinases like MAPK (Stabel and Parker, 1991; Monick et al., 2001). Ollivier et al. (1996) demonstrated that Protein Kinase-A (PKA) is involved in anti-inflammatory immune responses. The role of Phosphoinositide 3-kinase (PI-3K) in phagosome-lysozome fusion has been established (Vergne et al., 2003; Chua et al., 2004). Because of the critical role played by protein kinases in signal transduction pathways, we wished to test if they played any role in the regulation of HDAC1 expression. Toward that we inhibited MAPK-ERK, PKC, PKA, and PI-3K with specific inhibitors (Wymann et al., 1996; Pollack and Kawecki, 1997; Davies et al., 2000), and quantified the levels of HDAC1 in macrophages at 24 h PI. Of all the kinases, PKA seemed to positively regulate HDAC1 expression during MTB infection while the other kinases did not seem to play any significant role (Figures 7A,C).

### Role of CREB and c-Jun on expression of HDAC1 in MTB-infected macrophages

The ultimate function of many signal transduction pathways is the activation of transcription factors which enter the nucleus and bind to promoter sequences of specific genes to activate their transcription. We investigated the role of key transcription factors in the regulation of *HDAC1* expression upon MTB infection. Toward this we knocked down some important transcriptional regulators such as CREB, c-JUN, and SOX-5 (Lamph et al., 1990; Zheng et al., 2002; Wen et al., 2010) in macrophages prior to infection with MTB. CREB directly inhibits pro-inflammatory responses (Wen et al., 2010) and therefore we hypothesized that this CREB-mediated suppression of pro-inflammatory response could be mediated by the high levels of HDAC1 in macrophages infected with live, virulent MTB. In addition, recently it has been demonstrated that BCG suppresses epithelial proinflammatory responses, and that c-Jun level increases in bronchial epithelial cells at an early time point following BCG infection (Lutay et al., 2014). These observations prompted us to investigate whether there is a direct correlation between CREB, c-Jun and HDAC1 in regulating immune response during MTB infection. Interestingly, we found that knockdown of *CREB* and *c-Jun* resulted in significant reduction in the levels of HDAC1 in macrophages infected with MTB while the reduction was marginal in the case of *SOX-5* (Figures 7B,D). The efficiency of knockdown of each transcription factors were shown in the Supplementary data (Figure S6).

### c-Jun is recruited onto *HDAC1* promoter in macrophages during MTB infection

**Figure 8:**
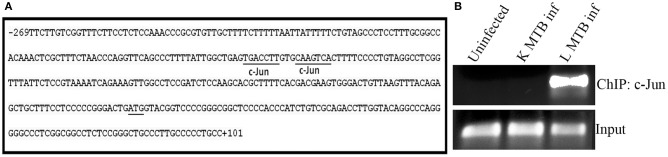
**Recruitment of c-Jun on *HDAC1* promoter in macrophages during MTB infection. (A)** Sequence analysis of *HDAC1* promoter region (−269 to +101 retrieved from Eukaryotic Promoter Database) revealed the presence of two putative consensus binding sequence (dissimilarity < 15%) for transcription factor c-Jun. c-Jun binding sites, PCR- amplified region, and TSS are indicated; **(B)** ChIP analysis for the recruitment of c-JUN on *HDAC1* promoter. Data from one of three independent experiments are shown.

Since CREB and c-Jun were found to play a role in the expression of *HDAC1*, we tested if these transcriptional regulators bind to the *HDAC1* promoter. Using ALGGEN PROMO (Messeguer et al., 2002), an online tool to predict binding sites of transcription factors, we found that *HDAC1* promoter has two putative binding sites for c-Jun (Figure 8A), and no binding site for CREB. To verify this, we carried out a ChIP with c-Jun antibodies followed by a PCR which amplified a 182 bp sequence (Figure 7B) of the *HDAC1* promoter containing the top ranked putative binding site. Interestingly, c-Jun occupancy on *HDAC1* promoter was observed only in macrophages infected with live MTB H37Rv whereas c-Jun was not recruited to the same in uninfected macrophages and those infected with heat-killed MTB (Figure 8B).

### Survival of MTB is negatively impacted in macrophages deficient in HDAC1

**Figure 9:**
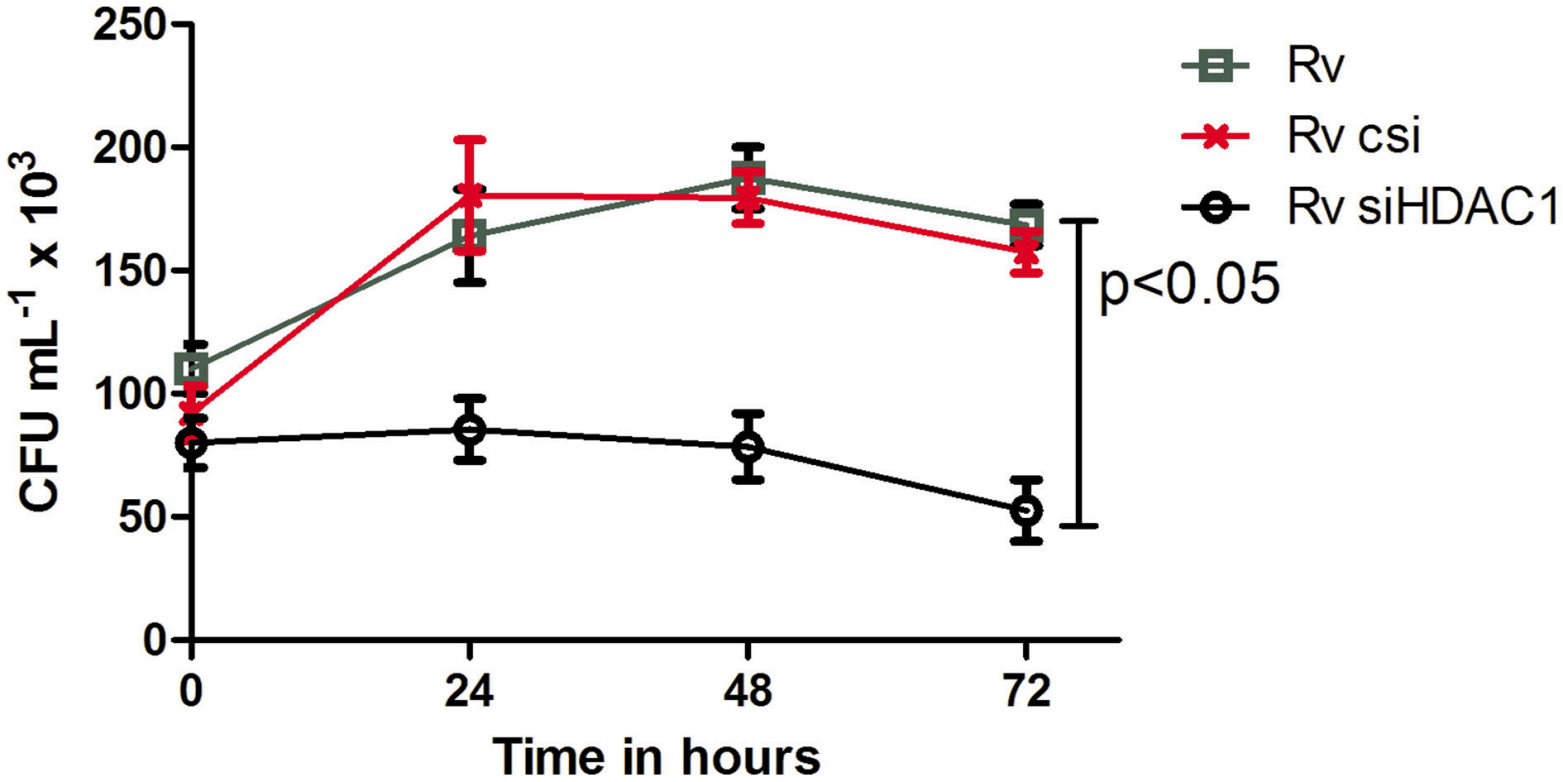
**Survival of MTB decreases when HDAC1 is knocked down in macrophages**. THP-1 monocytes (1 × 10^6^ cells) seeded in a 6-well plate were treated with scrambled siRNA and siHDAC1, and infected with MTB. Macrophages not treated with siRNA but infected with MTB were also kept as control. The number of bacteria recovered from the macrophages 4 h after infection was considered as the number of bacteria that gained entry into the macrophages (and the number at 0 h for studying the viability inside macrophages); and subsequent isolation of bacilli from the macrophages was carried out at 24, 48, and 72 h. The number of viable bacilli in each of the wells was assayed by plating on a 7H10 agar plates and incubating the plates at 37°C, and counting the colony forming unit (cfu). Data shown here are the mean ± SD of three independent experiments.

Since we observed a direct correlation between infection with live, virulent MTB and the levels of HDAC1 in macrophages, we wished to test whether HDAC1 plays a role in the survival of the bacteria inside the macrophages. Toward this, we knocked down HDAC1 in macrophages and infected them with live MTB H37Rv. Intracellular cfu were enumerated by lysing the infected macrophages at different time points (0, 24, 48, and 72 h PI) and plating them on Middlebrook 7H10 agar. Macrophages treated with scrambled siRNA and infected with live virulent MTB served as control. We observed a significant reduction in the number of cfu in the HDAC1-deficient macrophages (Figure 9).

## Discussion

In susceptible hosts, MTB succeeds in subverting host immune response pathways by epigenetic modifications leading to its survival and replication within the macrophages. In this study we observed that 24 h after infection, there was a dramatic upregulation of HDAC1, and a concomitant decrease in the levels of acetylation of histone H3 in macrophages infected with live virulent MTB H37Rv. However we did observe that at 12 h PI, the HDAC1 levels were low and the H3 acetylation was correspondingly high. These fundamentally opposite phenomena suggest that the responses at 12 h could be an initial reaction of macrophage to the presence of any infectious agent in its attempt to upregulate host immune genes, because the same response was observed when the cells were treated with heat-killed virulent MTB also. However, the opposite reaction at 24 h suggests that it could be directed by live virulent MTB to counter this assault from the host for its intracellular survival. It has been reported that during the initial period of MTB infection, bacterial numbers decline as macrophage attempts to eradicate the infection whereas at the later stage bacterium adapts to, and tries to modulate its environment to survive inside the macrophage (Rohde et al., 2012). Microarray analyses of MTB during intracellular infection have shown that several bacterial genes are upregulated during specific stages of infection which help the bacterium adapt to intracellular life (Rohde et al., 2012). The inactivation of transcriptional regulators in the killed MTB, as against the functional ones in live virulent MTB, can be the reason for the difference in responses in macrophages (Zahrt and Deretic, 2001). The observation that HDAC1 levels decrease in macrophages treated with LPS or infected with nonpathogenic MTB H37Ra clearly indicates that this is a mechanism executed by live virulent pathogen to survive inside the macrophages. Transcriptomic studies by Koo et al. (2012) identified HDAC1 as one of the genes activated after infection of bone marrow-derived macrophages with MTB. Here we demonstrated that the levels of HDAC1 protein in the macrophages indeed increase after infection with live, virulent MTB whereas they decrease in macrophages infected with live, non-virulent MTB H37Ra, and even in those treated with heat-killed MTB H37Rv or LPS. The enhancement of HDAC1 levels appears to be an ingenious strategy employed by the intracellular MTB to prevent the host defense gene expression by deacetylating histones in the promoters of those genes to facilitate its survival inside the host. Intracellular pathogens are known to modulate host gene expression through chromatin modification by HDACs. For instance, infection by *Anaplasma phagocytophilum*, a Gram-negative bacterium that resides in granulocytes and causes granulocytic anaplasmosis in humans, upregulates HDAC expression and the bacterial AnkA protein was shown to interact with gene regulatory regions of host chromatin silencing host cell defense genes (Garcia-Garcia et al., 2009a,b). The secreted Listerial virulence factor, LntA, targets the chromatin repressor BAHD1, a recruiter of HDAC1 and HDAC2 in the host cell nucleus, to modulate pro-inflammatory cytokine production (Lebreton et al., 2011). Infection by *Moraxella catarrhalis*, one of the reasons for exacerbation of chronic obstructive pulmonary disease, induces reduction in the levels of expression and activity of HDAC1 and HDAC2 in bronchial epithelial cells. However, in our study we observed that at 24 h PI, only HDAC1, and not HDAC2, is upregulated significantly in macrophages infected with MTB. Different classes of HDACs are known to have different biological activities as they differ in cellular localization and in their presence in transcription complexes regulating the expression of a variety of genes (Jones et al., 2001; Hagelkruys et al., 2014). Wang et al. (2005) showed that inhibition of IFN-γ induced expression of HLA-DRα and HLA-DRβ genes upon *Mycobaterium avium* infection can be restored by HDAC inhibitors. No difference in HDAC1 and HDAC2 gene expression was observed, however they found an increase in the expression of *Sin3A* co-repressor required by class-1 HDACs for its activity. This probably suggests that the type of HDAC expressed following infection is specific to each pathogen. Our experiments with primary macrophage cells further prove that the increased level of HDAC1 observed is due to the infection by virulent M. tuberculosis irrespective of the cells used. In view of these observations, we hypothesized that the significant upregulation of HDAC1 in macrophages, and the consequent decrease in the histone H3 acetylation, could be responsible for the downregulation of the expression of a number of host immune genes following MTB infection.

Th1 responses are downregulated during MTB infection (Kaplan et al., 1996; Magombedze et al., 2014). From our results, it is possible to speculate that one of the possible reasons for the downregulation of this immune response could be an HDAC1-mediated transcriptional repression of *IL-12B* gene. Expression of genes in different cell types has been shown to be negatively affected by disruption of HDAC activity with inhibitors or by siRNA (Bryant et al., 2004; Galán and Cossart, 2005; Zupkovitz et al., 2006). We showed the involvement of HDAC1 in the downregulation of *IL-12B* unequivocally by inhibiting HDAC activity by TSA, and by *HDAC1* knockdown. Kobayashi et al. (2012) reported that inhibitory effect of IL-10 on LPS-induced *IL-12B* expression in intestinal macrophages is regulated by HDAC3 (Kobayashi et al., 2012). However, we observed only a marginal increase in the expression of *HDAC3*, (Figure S7) in comparison with *HDAC1*, in THP-1 derived macrophages upon MTB infection, although its role in regulating *IL-12* or other genes in macrophages upon MTB infection cannot be ruled out. IL-12 is the key cytokine that induces differentiation of naive Th0 subpopulation of T-cells into Th1 cells, and thus plays a crucial role in linking innate and acquired immune responses, allowing phagocytic cells to facilitate the development of cell-mediated immunity to intracellular bacterial, viral, parasitic, and fungal pathogens (Romani et al., 1994; Szabo et al., 1995; Cooper et al., 1997). Co-transfection of 293T human embryonic kidney epithelial cells with *IL-12B* and *HDAC1* genes showed that HDAC1 has a repressing effect on the activity of *IL-12B* promoter (Lu et al., 2005), and corroborates our similar observation in MTB-infected macrophages. Interestingly, our study revealed that the HDAC1 recruitment at the *IL-12B* promoter is brought about only by metabolically active virulent MTB. This suggests that while inside the macrophages MTB may release virulence factors that may directly or indirectly facilitate the recruitment of host HDACs to the appropriate promoters. Although we showed HDAC1 recruitment on *IL-12B* promoter, the same phenomenon occurring on promoters of other defense genes following MTB infection is an interesting possibility. How HDAC1 is recruited to the promoter of these genes during infection, however, requires further investigation.

**Figure 10:**
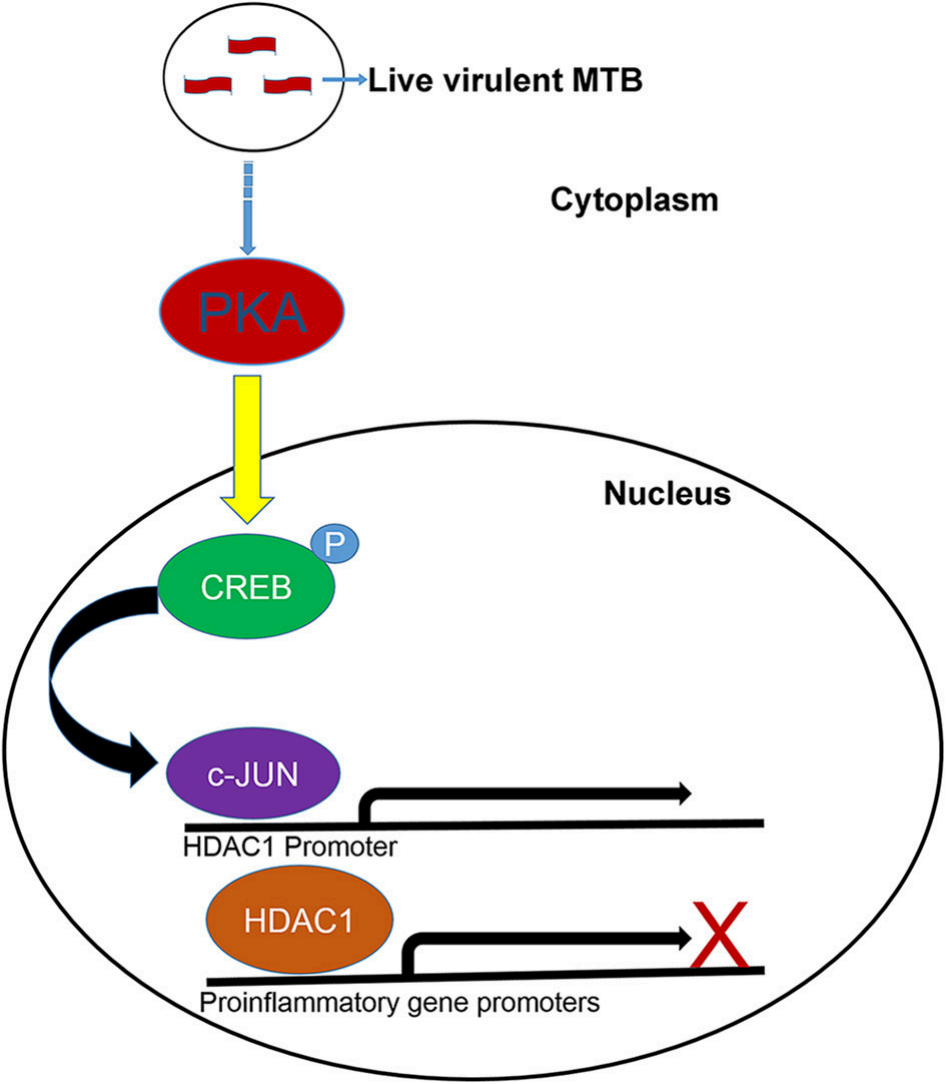
**Proposed model for the upregulation of HDAC1 in macrophages infected with MTB**. PKA phosphorylates CREB leading to the transcriptional activation of *c-Jun* gene. c-Jun is recruited onto the *HDAC1* promoter of macrophages infected with live, virulent MTB. HDAC1 is recruited to the promoters of proinflamatory genes resulting in transcriptional repression.

In this study we tried to delineate the mechanism behind HDAC1 upregulation in macrophages upon MTB infection. Treatment of cells with kinase inhibitors indicated that the Protein kinase-A (PKA) could be playing a role, directly or indirectly, in the regulation of HDAC1 expression in macrophages during MTB infection. It has been shown that regulation of activity of class-I HDACs involves their direct association with kinases (Saha and Pahan, 2006). We wished to probe into the connection between PKA and the upregulation of HDAC1 expression. Interestingly, employing siRNA, we have proven that cyclic-AMP response element-binding protein (CREB) and c-Jun are involved in the regulation of HDAC1 expression. CREB has a role in regulating several immune-related genes such as IL-2, IL-6, IL-10, and TNF-α (Wen et al., 2010). Li and Verma showed that phosphorylated CREB prevents NF-κB activation by blocking the binding of CREB binding protein (CBP) to the NF-κB complex (Li and Verma, 2002). CREB is an activator of anti-inflammatory responses in macrophage cells (Martin et al., 2005) and it has been shown that c-Jun is upregulated in macrophages infected with BCG (Lutay et al., 2014). Lamph et al. (1990) have demonstrated that the transcriptional activity of *c-Jun* promoter is repressed by CREB and this repression is alleviated only when CREB is phosphorylated by the catalytic subunit of PKA (Lamph et al., 1990). Many studies have shown that following mycobacterial infection, levels of intracellular cAMP, a potent inhibitor of pro-inflammatory cytokines, go up many folds (Serezani et al., 2008; Agarwal et al., 2009; Pelly et al., 2012). Agarwal et al. (2009) have shown cAMP-induced upregulation of PKA in macrophages upon MTB infection (Agarwal et al., 2009). Interestingly, they have shown that bacterially-derived cAMP is released into the cytoplasm of macrophage with the aid of mycobacterial adenylate cyclase (Rv0386) and this resulted in the activation of PKA (Agarwal et al., 2009) and probably leads to the phosphorylation of CREB. This enhanced the survival of MTB within the macrophages, representing a mechanism by which CREB promotes bacterial survival. As PKA functions upstream of CREB (Quinn, 1993; Du et al., 2000; Tacke et al., 2005), our results suggested that PKA-activated CREB might be involved in regulating MTB-mediated *HDAC1* expression. Therefore, we construed that the cAMP burst resulting from MTB infection probably activates PKA which in turn phosphorylates CREB leading to the transcriptional activation of *c-Jun* gene. While exploring the link between c-Jun and *HDAC1* expression, we observed that *HDAC1* promoter possesses two putative binding sites for c-Jun (**TGAC**CTT/CAA**GTCA**) (Li et al., 2011). Thus, it is highly likely that binding of c-Jun to *HDAC1* promoter is essential for the enhancement of *HDAC1* expression during MTB infection. Chasing this logic with ChIP and PCR analyses, we could demonstrate that c-Jun is recruited only onto the *HDAC1* promoter of macrophages infected with live MTB H37Rv, and not onto that in uninfected macrophages and those infected with heat-killed MTB H37Rv (Figure 10). This requirement for c-Jun binding to its promoter possibly explains the reason for the downregulation of *HDAC1* when *c-Jun* is knocked down in infected macrophages.

IL-12 is a cytokine that plays a crucial role in eliciting Th1 response following MTB infection (Cooper et al., 1993, 1995). Utsugi et al. (2003) have reported that *IL-12B* is downregulated in THP-1 macrophages as a result of increased phosphorylation of c-Jun (Utsugi et al., 2003). Here we were able to show that in macrophages infected with live MTB H37Rv, HDAC1 is recruited to the promoter of *IL-12B* where histone H3 is hypoacetylated. Put together, data from these two studies and our own study suggest that *IL-12B* expression in MTB-infected macrophages is probably downregulated by the recruitment of HDAC1 (which in turn was upregulated by the recruitment of c-Jun) onto the *IL-12B* promoter. We found that the expression of HDAC1 was marginally affected by the knockdown of *SOX5*. SOX5 is a transcriptional regulator reported to control L-type Ca^2+^ channels (Zheng et al., 2002). Knockdown of *SOX5* in macrophages perhaps negatively impacted intracellular calcium homeostasis thereby affecting the downstream pathways. Further studies are required to understand the role of SOX5 in *HDAC1* expression.

Interestingly, intracellular survival of MTB seems to depend on HDAC1 activity as the number of viable MTB was significantly reduced in cells deficient in the same. This observation substantiates the recent predictions that HDAC inhibitors can be potential therapeutic agents to control MTB infection (Choi et al., 2013; Zumla et al., 2015). However, how HDAC1 contributes to the survival of intracellular MTB is not clear at present. Direct involvement of IL-12 in this phenomenon seems less likely, and therefore it will be interesting to explore how HDAC1 plays its role in the intracellular survival of MTB.

Thus, our results indicate an interesting mechanism for immune subversion by virulent MTB. Modulation of host gene expression by interfering with the kinetics of acetylation and deacetylation of histones could tailor the responses in susceptible hosts in favor of the pathogen thus giving sufficient time to the bacterium to establish persistent infection and thwart pro-inflammatory responses launched by the host. Delineation of mechanisms regulating acetylation and deacetylation during MTB infection will further our understanding of the molecular interactions between man and one of the most successful bacterial pathogens. In addition, our results corroborate the proposition that host-directed therapies can become a promising strategy to treat TB.

## Author contributions

RAK supervised the project. AC and RAK designed the experiments, analyzed data, and wrote the manuscript with contributions from all the co-authors. AC performed most of the experiments and statistical analysis. AC and CA performed siRNA experiments. KN provided reagents and advice in siRNA experiments. LJ and SM extended technical advice and support. LJ, KN, SM, and RAK edited the manuscript.

### Conflict of interest statement

The authors declare that the research was conducted in the absence of any commercial or financial relationships that could be construed as a potential conflict of interest.
