# Supplementary material for: Mycobacterium tuberculosis Infection Induces HDAC1-Mediated Suppression of IL-12B Gene Expression in Macrophages
Source: Front Cell Infect Microbiol. 2015 Dec 2;5:90. doi: 10.3389/fcimb.2015.00090 (PMC4667035; doi:10.3389/fcimb.2015.00090)
Supplement: Supplementary file 1 [file DataSheet1.DOC]

***Mycobacterium tuberculosis* infection induces HDAC1-mediated suppression of *IL-12B* gene expression in macrophages.**

Aneesh Chandran^1^, Cecil Antony^2^, Leny Jose^1^, Sathish Mundayoor^1^, K. Natarajan^2^ and R. Ajay Kumar^1*^.

^1^Mycobacterium Research Group, Pathogen Biology Program, Rajiv Gandhi Centre for Biotechnology, Thycaud P.O. Thiruvananthapuram 695014, India.

^2^Infectious Diseases Laboratory, Dr. B. R. Ambedkar Centre for Biomedical Research, University of Delhi, Delhi 110007, India.


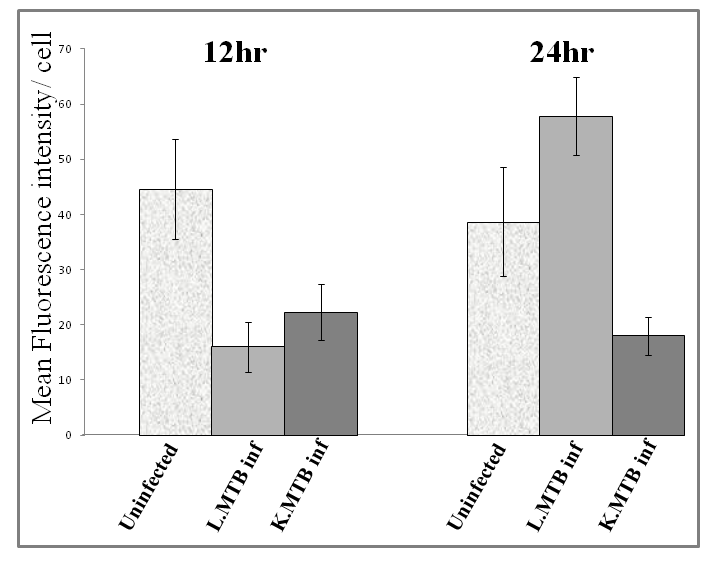
**Supplementary data**

Figure S1. Immunofluorescence quantification of HDAC1 at 12hr and 24 hr (from Figs 2A and 2B).  Image analysis was performed using ImageJ v3.91 software (<http://rsb.info.nih.gov/ij>).   Ten cells from each field were randomly selected from three independent experiments for measuring mean fluorescence intensity. For each cell, binary image masks were created of HDAC1 positive staining to define regions of interest (ROI) for analysis.


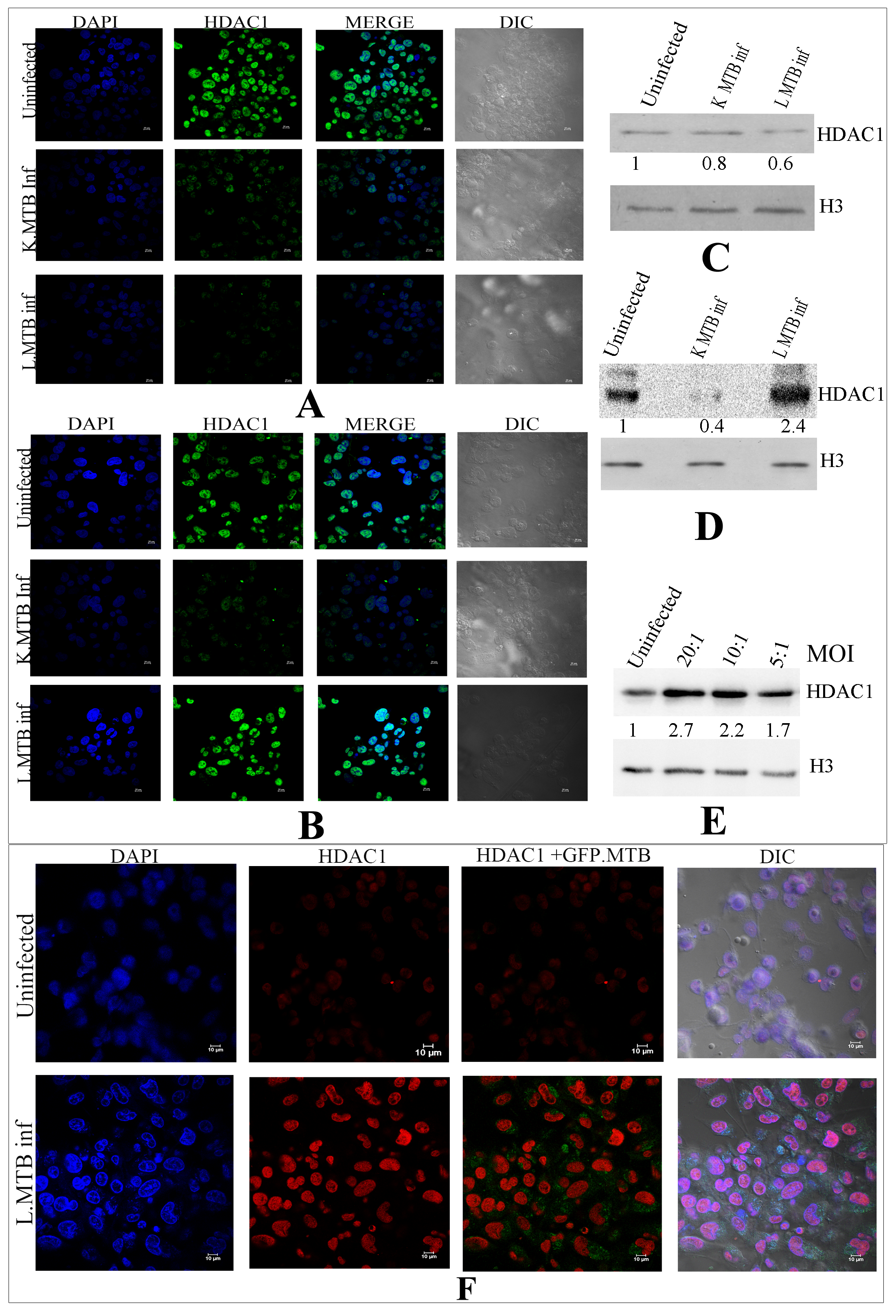


**Figure S2. Immunocytochemical images show the expression of HDAC1 (red) in the presence of intracellular MTB expressing GFP.** HDAC1 level increases upon MTB infection at 24 hr PI.

**
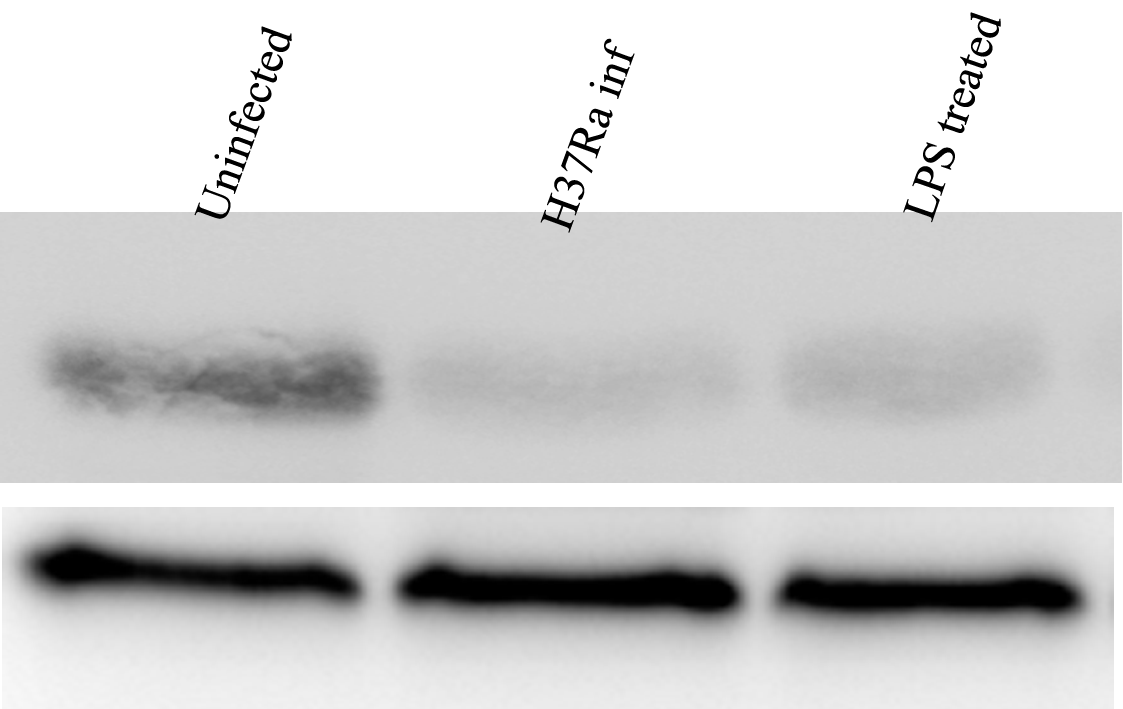
**

**HDAC1**

**H3**

**Figure S3. Status of HDAC1 in macrophages during different infection conditions.** HDAC1 level decreases in macrophages upon non-pathogenic MTB H3Ra infection and upon treatment with LPS at 24 hr PI.

**
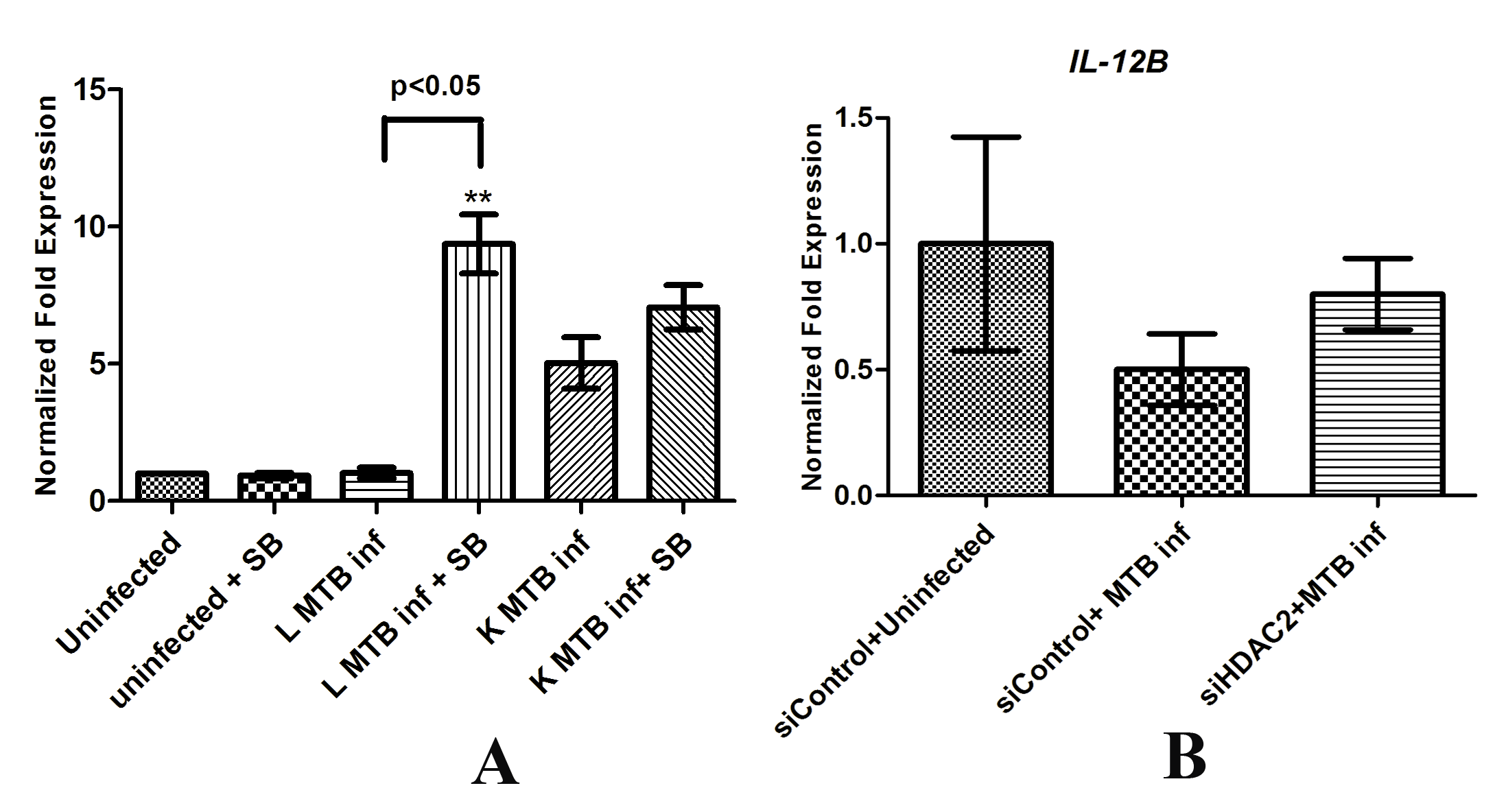
**

**Fig S4: Status of HDAC1 in MTB-infected macrophages in the presence of HDAC inhibitor and during HDAC2 knockdown (A)** *IL-12B* expression is upregulated in MTB-infected macrophages upon HDAC inhibition with sodium butyrate (B) No significant change in *IL-12B* expression in MTB-infected macrophages when HDAC2 is knocked down.


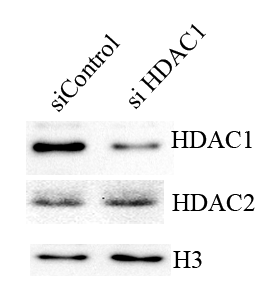

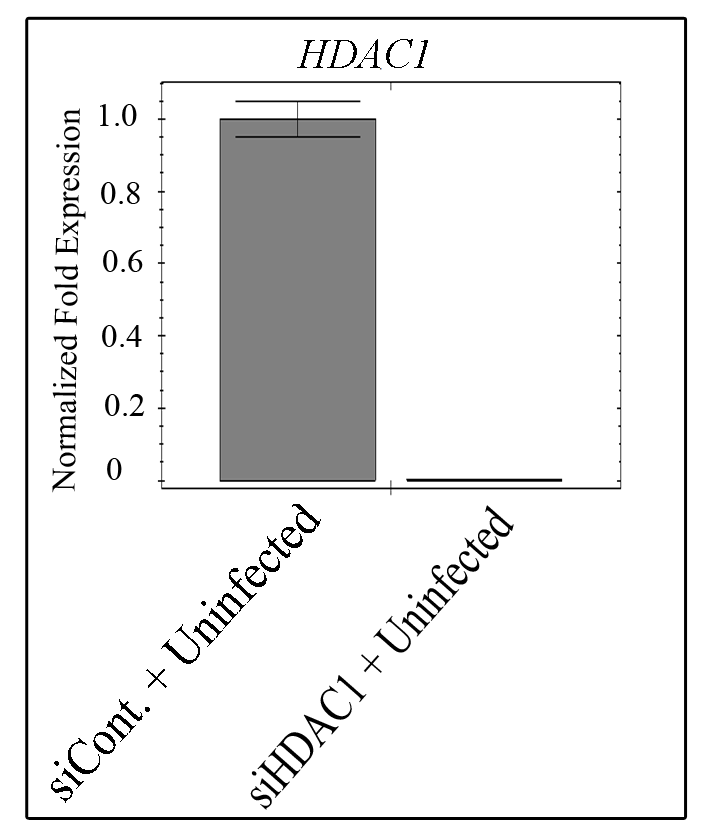


A B

**Figure S5**. **Efficiency and specificity of siHDAC1 knockdown**. (A) qPCR analysis shows more than 90% decrease in the *HDAC1* expression upon silencing of macrophages with siHDAC1; (B) Western blot shows more than 70% decrease in the HDAC1 level upon silencing of macrophages with siHDAC1. Probing with HDAC2 antibody confirms the specificity of HDAC1 silencing.

**
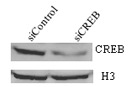

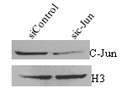

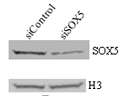
**

A B C

**Figure S6. Efficiency of knockdown by western blot** (A) CREB (B) c-JUN and (C) SOX5


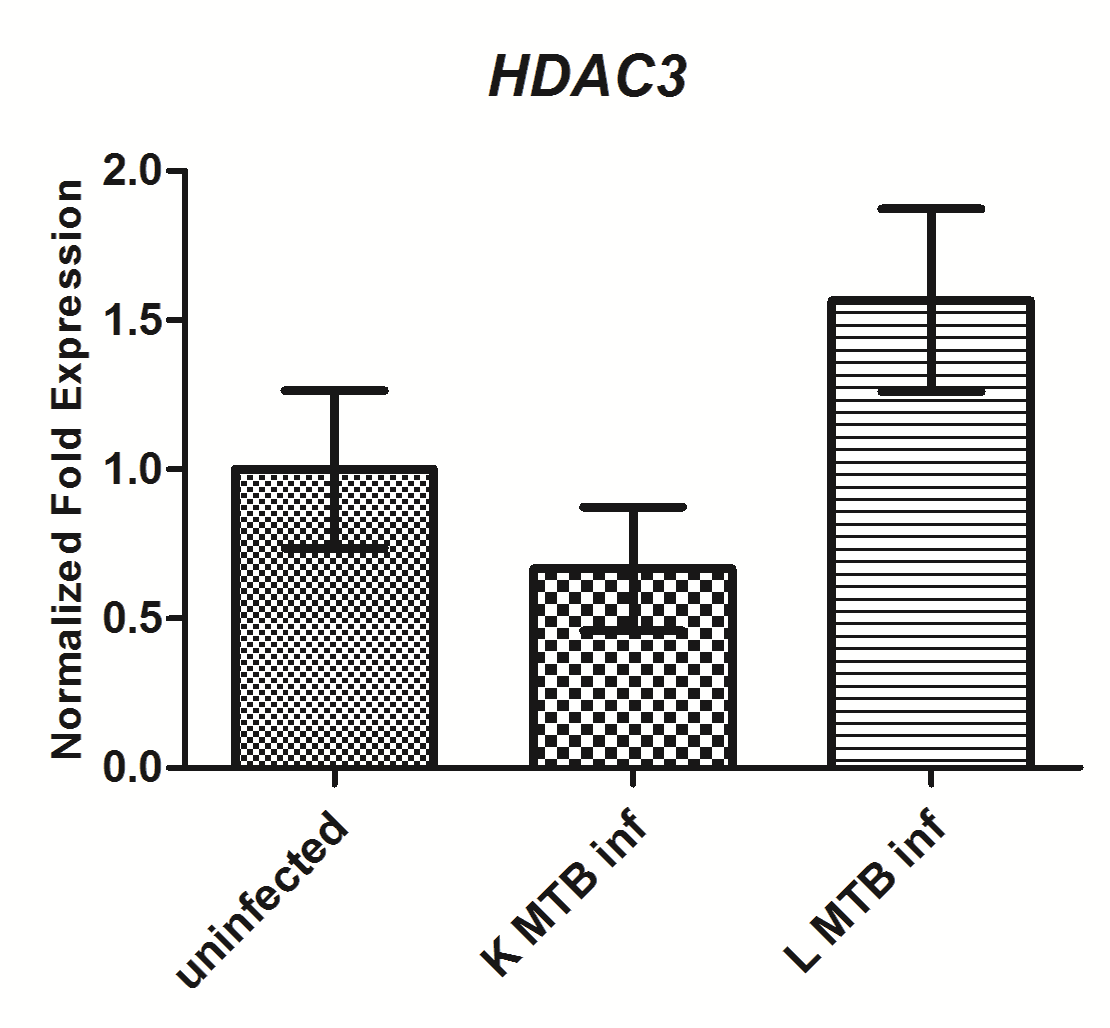


**Figure S7. Status of expression of *HDAC3* in macrophages upon MTB infection.** *HDAC3* expression at 24 hr PI. Each result represents the mean ± SD of data from three experiments.

**Table 1: Promoter primers**

| **SL**  **NO** | **PROMOTER** | **FORWARD PRIMER** 5'-3' | **REVERSE PRIMER** 5'-3' |
| --- | --- | --- | --- |
| 1 | *HDAC1*  (+13 to -169) | ATTGGCTGAGTGACCTTGTG | GGGACCGTACCATCA |
| 2 | *IL-12B*  (+12 to -539) | TCTTTCTTCTGCTGCTGTTG | AGCCAAGATGGGTGGTAAA |
|  |  |  |  |

**Table 2: Gene primers**

| **SL.NO** | **GENE** | **FORWARD PRIMER 5'-3'** | **REVERSE PRIMER 5'-3'** |
| --- | --- | --- | --- |
| 1 | *HDAC1* | CATCTCCTCAGCATTGGCTT | CGAATCCGCATGACTCATAA |
| 2 | *HDAC2* | ATGAGGCTTCATGGGATGAC | ATGGCGTACAGTCAAGGAGG |
| 3 | *STAT4* | AATAATCCTCCACCTGCCACATTG | CTGAGTTAAGACCACGACCAACG |
| 5 | *GAPDH* | AATGAAGGGGTCATTGATGG | AAGGTGAAGGTCGGAGTCAA |
| 6 | *IL-12B* | CAGTGGAGTGCCAGGAGGACAGTG | AAGTTCTTGGGTGGGTCAGGTTTG |
| 7 | *HDAC3* | GCTGGGTGGTGGTGGTTATAC | ATCTGGATGAAGTGTGAAGTCTGG |
